# In vitro activities of natural products against oral *Candida *isolates from denture wearers

**DOI:** 10.1186/1472-6882-11-119

**Published:** 2011-11-26

**Authors:** Cristina Marcos-Arias, Elena Eraso, Lucila Madariaga, Guillermo Quindós

**Affiliations:** 1 Laboratorio de Micología Médica, Departamento de Inmunología, Microbiología y Parasitología, Facultad de Medicina y Odontología, Universidad del País Vasco/Euskal Herriko Unibertsitatea, Bilbao, Spain

## Abstract

**Background:**

*Candida*-associated denture stomatitis is a frequent infectious disease. Treatment of this oral condition is difficult because failures and recurrences are common. The aim of this study was to test the in vitro antifungal activity of pure constituents of essentials oils.

**Methods:**

Eight terpenic derivatives (carvacrol, farnesol, geraniol, linalool, menthol, menthone, terpinen-4-ol, and α-terpineol), a phenylpropanoid (eugenol), a phenethyl alcohol (tyrosol) and fluconazole were evaluated against 38 *Candida *isolated from denture-wearers and 10 collection *Candida *strains by the CLSI M27-A3 broth microdilution method.

**Results:**

Almost all the tested compounds showed antifungal activity with MIC ranges of 0.03-0.25% for eugenol and linalool, 0.03-0.12% for geraniol, 0.06-0.5% for menthol, α-terpineol and terpinen-4-ol, 0.03-0.5% for carvacrol, and 0.06-4% for menthone. These compounds, with the exception of farnesol, menthone and tyrosol, showed important in vitro activities against the fluconazole-resistant and susceptible-dose dependent *Candida *isolates.

**Conclusions:**

Carvacrol, eugenol, geraniol, linalool and terpinen-4-ol were very active in vitro against oral *Candida *isolates. Their fungistatic and fungicidal activities might convert them into promising alternatives for the topic treatment of oral candidiasis and denture stomatitis.

## Background

Denture stomatitis is a common inflammatory reaction in denture-wearing patients, characterized by an erythematous inflammation of mucosal areas covered by dentures. Although this clinical entity is multifactorial, *Candida albicans *is the major etiological agent [1]. Moreover, other species of *Candida*, such as *C. glabrata*, *C. tropicalis*, *C. krusei*, *C. parapsilosis *and *C. dubliniensis *have been isolated from patients with denture stomatitis [2-4]. Different treatments have been proposed for *Candida*-associated denture stomatitis. However, there is a low number of antifungal agents and therapy can induce side effects, resistance and/or recurrence [1,5]. Thus, new therapeutic strategies are necessary and natural products can play an important role in the treatment as some of them can be included in mouthrinses or tooth pastes. Among natural products, essential oils are promising therapeutic tools for oral infections. These oils are complex mixtures of volatile compounds obtained from plants, such as the terpenoids, with antioxidant and antimicrobial properties against a wide range of pathogens, including *Candida albicans *and dermatophytes [6-9].

The aim of the current study was to investigate the in vitro activity of eight terpenic derivatives (carvacrol, farnesol, geraniol, linalool, menthol, menthone, terpinen-4-ol, and α-terpineol), a phenylpropanoid (eugenol) and a phenethyl alcohol (tyrosol) against oral *Candida *isolates from patients suffering from denture stomatitis.

## Methods

### Microorganisms

A total of 38 oral isolates were tested, including 10 *C. albicans*, 10 *C. glabrata*, 10 *C. tropicalis*, 5 *C. guilliermondii*, and 1 isolate each of *C. parapsilosis*, *C. dubliniensis *and *C. krusei*. The clinical isolates were randomly selected from those recovered from a prospective study in denture wearers attending the Odontology clinics at the Universidad del País Vasco/Euskal Herriko Unibertsitatea, Bilbao (Spain). Demographic and clinical characteristics of these patients have been published elsewhere [2]. The institutional review board of Universidad del País Vasco/Euskal Herriko Unibertsitatea approved the study and informed consent was obtained in each patient included in the study. There were also studied 10 type strains from the American Type Culture Collection (ATCC) and the National Collection of Pathogenic Fungi (NCPF) including *C. albicans *ATCC 90028, *C. albicans *NCPF 3153, *C. glabrata *ATCC 90030, *C. glabrata *NCPF 3203, *C. dubliniensis *NCPF 3949, *C. guilliermondii *NCPF 3099, *C. krusei *ATCC 6258, *C. parapsilosis *ATCC 22019, *C. tropicalis *NCPF 3111, and the *C. albicans *NCPF 3153 hypha-defective mutant Ca2 (kindly donated by Professor Antonio Cassone, Istituto Superiore di Sanità, Rome, Italy). Isolates were identified by conventional mycological methods, such as the germ tube test in serum, microscopic morphology, chlamydoconidia production in corn meal agar with Tween 80, and carbon source assimilation with the commercial kit ID 32 C (bioMérieux, France) [10].

### Terpenes

**Figure 1:**
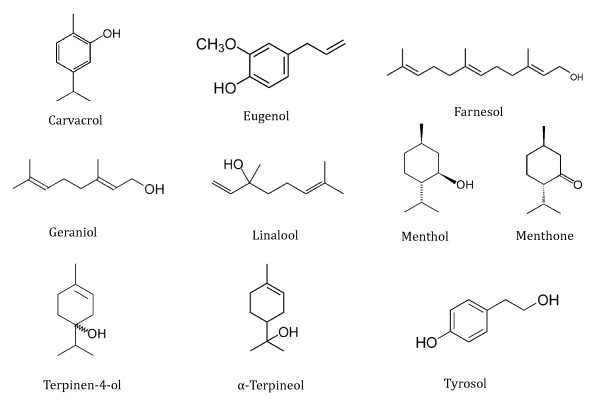
**Formulae of the eight terpenic derivates (carvacrol, farnesol, geraniol, linalool, menthol, menthone, terpinen-4-ol, and α-terpineol), the phenylpropanoid (eugenol) and the phenethyl alcohol (tyrosol) studied**.

All evaluated compounds were purchased from Sigma (Sigma-Aldrich, USA) and included: carvacrol (5-isopropyl-2-methylphenol) from *Satureja hortensis*, eugenol (4-allyl-2-methoxyphenol) from *Pimenta dioica*, farnesol ((2*E*,6*E*)-3,7,11-trimethyldodeca-2,6,10-trien-1-ol) from *Vachellia farnesiana*, geraniol (3,7-dimethylocta-2,6-dien-1-ol) from *Rosa damascena*, linalool (3,7-dimethylocta-1,6-dien-3-ol) from *Coriandrum sativum*, menthol ((1*R*,2*S*,5*R*)-2-isopropyl-5-methylcyclohexanol) from *Mentha piperita*, menthone ((2*S*,5*R*)-trans-2-isopropyl-5-methylcyclohexanone) from *Mentha piperita*, terpinen-4-ol (4-isopropyl-1-methyl-1-cyclohexen-4-ol) from *Melaleuca alternifolia*, α-terpineol (2-(4-methyl-1-cyclohex-3-enyl) propan-2-ol) from *Artemisia annua*, and tyrosol (4-(2-hydroxyethyl)phenol) from *Olea europaea *(Figure 1). The compounds were prepared as stock solution of 16% (volume/volume -v/v-) in RPMI 1640 medium and 0.1% (v/v) Tween 80 (Sigma-Aldrich) [11].

### In vitro antifungal activity testing

The antifungal activities of terpenes and fluconazole (as a positive control) were determined by broth microdilution method using two fold serial dilutions in RPMI 1640 medium, as described in the document M27-A3 from the Clinical Laboratory Standards Institute (CLSI) for yeasts [12]. Briefly, chemical compounds were dissolved in RPMI 1640 medium buffered to pH 7.0 with 0.165 M morpholinepropanesulfonic acid. After shaking, 100 μl aliquots were added to the wells of 96-well microtiter plates with final concentrations ranging from 0.01% to 8% (v/v). To enhance the compounds solubility, Tween 80 was included in all assays at a final concentration of 0.05% (v/v) [11]. Fluconazole was prepared in pure water. Reference microdilution trays containing serial twofold dilutions of this drug were prepared in RPMI 1640 medium buffered to pH 7.0 with 0.165 M morpholinepropanesulfonic acid (MOPS) buffer in 96-well plates, and stored at -70°C for no longer than 3 months.

Yeast inocula were prepared by growing the isolates on Sabouraud dextrose agar plates for 24 h at 37°C and adjusting to a final concentration between 1 × 10^3 ^and 5 × 10^3 ^cells/ml in sterile saline. A 100 μl suspension of each of the *Candida *strains was added to individual wells and cultivated at 37°C for 24-48 h. Fluconazole concentrations, when reconstituted with the yeast suspensions, ranged from 0.12 to 64 μg/ml. Chemical-free and yeast-free controls were included.

### Determination of minimum inhibitory and minimum fungicidal concentrations

The minimum inhibitory concentration (MIC) of the evaluated compounds for each isolate was determined according to the CLSI M27-A3 methodology. The MIC was defined as the lowest concentration that produced a prominent decrease of fungal growth (inhibition ≥ 50%) compared with growth control. The minimum concentration of drug that inhibited 90% and 50% of the isolates tested was defined as MIC_90 _and MIC_50_, respectively.

The MIC of fluconazole was read as the lowest concentration that produced a prominent decrease of growth (MIC_2_: inhibition ≥ 50%) relative to the control of growth, with no antifungal agent. Classification of isolates in terms of their susceptibilities to these antifungal agents was based on the MIC breakpoints recommended in the M27-A3 protocol and the M27-S3 supplement of the CLSI [12]. The MIC for susceptibility was ≤ 8 μg/ml, the MIC for susceptible-dose dependent was 16 to 32 μg/ml, and the MIC for resistance was ≥ 64 μg/ml. For quality control, *C. krusei *ATCC 6258, and *C. parapsilosis *ATCC 22019, were used.

Minimum fungicidal concentration (MFC) determinations were performed according to the modifications suggested by Cantón et al. [13]. Fungicidal activity was defined as a ≥ 99.9% reduction in the number of colony-forming units from the starting inoculum count, whilst fungistatic activity was defined as ≤ 99.9% reduction. The minimum concentration of drug that is fungicidal to 90% of the isolates was defined as MFC_90_.

### Statistical analysis

Unless otherwise specified, all tests were performed in triplicate in separate experiments. Descriptive statistics were used for MIC, MFC, the range and the geometric mean of these parameters. In vitro susceptibility testing data from 24 h and 48 h were compared for all natural compounds with the aim of detecting differences between both reading times. Comparisons between group values were performed by Student's t-test, being p < 0.05 regarded as statistically significant.

## Results

The MIC and MFC values of the 10 tested compounds against the 10 type strains studied at 24 h are summarized in Table 1. Table 2 shows MIC values of these compounds against oral isolates. The results confirmed the antifungal activity of almost all the tested compounds. The range of MICs at 24 h was 0.03-0.5% for carvacrol, 0.03-0.25% for the phenylpropanoid eugenol, 0.03-0.12% for geraniol, 0.03-0.25% for linalool, 0.06-0.5% for menthol, 0.06-4% for menthone, 0.06-0.5% for terpinen-4-ol, 0.06-0.5% for α-terpineol, and 0.25-32 μg/ml for fluconazole. Tween 80 at 0.05% (v/v) did not show antifungal activity against the microorganisms studied (data not shown). Moreover, MIC readings at 48 h did not differ markedly from readings at 24 h (p values were between 0.06 and 0.67). There were no differences over a dilution between the three measurements for each test, but the highest MIC was considered for calculations.

**Table 1:** In vitro antifungal activity of the ten natural compounds against reference strains at 24 h (%, v/v)

	MIC/MFC									
	Carvacrol	Eugenol	Farnesol	Geraniol	Linalool	Menthol	Menthone	Terpinen-4-ol	α-terpineol	Tyrosol
*Candida albicans *ATCC 90028	0.25/0.5	0.12/0.12	8/8	0.12/0.12	0.12/0.12	0.25/0.5	0.25/0.5	0.12/0.12	0.25/0.5	4/8
*Candida albicans *NCPF 3153	0.25/0.5	0.06/0.12	8/8	0.06/0.12	0.06/0.25	0.25/0.5	0.25/0.5	0.06/0.12	0.12/0.25	8/8
*Candida albicans *Ca2	0.25/0.5	0.03/0.12	1/8	0.06/0.12	0.06/0.12	0.12/0.5	0.12/0.5	0.06/0.12	0.12/0.25	4/8
*Candida glabrata *ATCC 90030	0.12/0.5	0.03/0.12	0.12/2	0.12/0.12	0.06/0.06	0.12/0.5	0.12/0.5	0.12/0.12	0.12/0.5	8/8
*Candida glabrata *NCPF 3203	0.25/0.25	0.03/0.12	0.12/0.5	0.06/0.12	0.12/0.25	0.25/0.5	0.25/0.5	0.06/0.12	0.12/0.5	8/8
*Candida dubliniensis *NCPF 3949	0.25/0.5	0.06/0.12	0.06/8	0.06/0.12	0.03/0.12	0.12/0.25	0.12/0.25	0.12/0.5	0.12/0.25	4/8
*Candida guilliermondii *NCPF3099	0.12/0.5	0.03/0.06	0.12/8	0.06/0.06	0.06/0.06	0.12/0.25	0.12/0.25	0.06/0.12	0.12/0.25	4/8
*Candida krusei *ATCC 6258	0.12/0.5	0.12/0.5	4/8	0.12/0.5	0.12/1	0.5/2	0.5/2	0.06/0.25	0.12/0.5	2/4
*Candida parapsilosis *ATCC 22019	0.06/0.5	0.06/0.12	8/8	0.06/0.25	0.06/0.5	0.06/0.25	0.06/0.25	0.06/0.12	0.06/0.5	1/2
*Candida tropicalis *NCPF3111	0.25/0.5	0.12/0.12	0.5/8	0.06/0.12	0.06/0.12	0.12/0.5	0.12/0.5	0.12/0.12	0.5/0.5	8/8

**Table 2:** In vitro antifungal activity of the ten natural compounds against oral *Candida *isolates at 24 h (%, v/v)*.

		Species (No. of isolates)							
		*Candida albicans *(10)	*Candida glabrata *(10)	*Candida tropicalis *(10)	*Candida guilliermondii *(5)	*Candida parapsilosis *(1)	*Candida dubliniensis *(1)	*Candida krusei *(1)	All *Candida *(38)
Carvacrol	MIC range	0.12-0.5	0.12-0.25	0.06-0.25	0.25	0.03	0.12	0.12	0.03-0.5
	MIC_50_/MIC_90_	0.25/0.5	0.12/0.25	0.12/0.25	0.25/-	-/-	-/-	-/-	0.25/0.25
	GM MIC	0.286	0.173	0.151	0.250	0.03	0.12	0.12	0.187
	MFC_90_	0.5	1	1	-	-	-	-	1
	
Eugenol	MIC range	0.06-0.25	0.03-0.25	0.06-0.12	0.06-0.25	0.06	0.06	0.12	0.03-0.25
	MIC_50_/MIC_90_	0.12/0.25	0.06/0.25	0.06/0.12	0.06/-	-/-	-/-	-/-	0.06/0.25
	GM MIC	0.122	0.080	0.079	0.106	0.06	0.06	0.12	0.092
	MFC_90_	0.5	0.25	0.25	-	-	-	-	0.5
	
Farnesol	MIC range	8	0.12-0.5	0.5-8	0.25-8	4	0.25	8	0.12-8
	MIC_50_/MIC_90_	8/8	0.25/0.5	8/8	8/-	-/-	-/-	-/-	8/8
	GM MIC	8	0.267	6.063	4	4	0.25	8	2.487
	MFC_90_	> 8	> 8	> 8	-	-	-	-	8
	
Geraniol	MIC range	0.06-0.12	0.03-0.12	0.03-0.06	0.06-0.12	0.03	0.06	0.12	0.03-0.12
	MIC_50_/MIC_90_	0.12/0.12	0.06/0.12	0.03/0.06	0.06/-	-/-	-/-	-/-	0.06/0.12
	GM MIC	0.097	0.060	0.042	0.079	0.03	0.06	0.12	0.065
	MFC_90_	0.25	0.25	0.25	-	-	-	-	0.25
	
Linalool	MIC range	0.03-0.25	0.03-0.12	0.06-0.25	0.06-0.12	0.03	0.06	0.06	0.03-0.25
	MIC_50_/MIC_90_	0.12/0.25	0.06/0.12	0.06/0.25	0.06/-	-/-	-/-	-/-	0.06/0.25
	GM MIC	0.141	0.064	0.086	0.079	0.03	0.06	0.06	0.085
	MFC_90_	0.5	0.5	0.25	-	-	-	-	0.5
	
Menthol	MIC range	0.25-0.5	0.12-0.5	0.06-0.12	0.12-0.25	0.06	0.12	0.12	0.06-0.5
	MIC_50_/MIC_90_	0.25/0.5	0.12/0.5	0.12/0.12	0.12/-	-/-	-/-	-/-	0.12/0.25
	GM MIC	0.287	0.185	0.112	0.161	0.06	0.12	0.12	0.170
	MFC_90_	1	1	1	-	-	-	-	1
	
Menthone	MIC range	0.12-4	0.06-2	0.06-1	0.25-1	0.06	0.25	1	0.06-4
	MIC_50_/MIC_90_	0.25/2	0.12/0.5	0.06/0.5	0.25/-	-/-	-/-	-/-	0.25/1
	GM MIC	0.534	0.229	0.113	0.435	0.06	0.25	1	0.260
	MFC_90_	8	4	8	-	-	-	-	8
	
Terpinen-4-ol	MIC range	0.06-0.5	0.06-0.12	0.06-0.12	0.06-0.25	0.06	0.12	0.12	0.06-0.5
	MIC_50_/MIC_90_	0.12/0.5	0.12/0.12	0.06/0.06	0.12/-	-/-	-/-	-/-	0.12/0.5
	GM MIC	0.213	0.097	0.064	0.140	0.06	0.12	0.12	0.112
	MFC_90_	0.5	0.5	0.5	-	-	-	-	0.5
	
α-terpineol	MIC range	0.12-0.5	0.12-0.25	0.06-0.25	0.12-0.5	0.06	0.12	0.06	0.06-0.5
	MIC_50_/MIC_90_	0.25/0.5	0.25/0.25	0.25/0.25	0.25/-	-/-	-/-	-/-	0.25/0.5
	GM MIC	0.307	0.186	0.201	0.248	0.06	0.12	0.06	0.210
	MFC_90_	1	1	1	-	-	-	-	1
	
Tyrosol	MIC range	1-8	0.5-8	0.5-8	1-8	1	4	1	0.5-8
	MIC_50_/MIC_90_	1/8	4/8	4/8	8/-	-/-	-/-	-/-	4/8
	GM MIC	2.462	3.249	3.482	3.482	1	4	1	2.934
	MFC_90_	8	> 8	> 8	-	-	-	-	8
	
Fluconazole*	MIC range	0.25-0.5	0.5-32	0.25-2	2-16	1	0.12	64	0.25-32
	MIC_50_/MIC_90_	0.5/0.5	8/16	1/2	2/-	-/-	-/-	-/-	1/16
	GM MIC	0.435	6.063	0.812	4.595	1	0.12	64	1.576
	MFC_90_	-	-	-	-	-	-	-	-

* Fluconazole concentrations are in μg/ml

The most actives compounds against *C. albicans *were eugenol, geraniol, linalool and terpinen-4-ol with MIC_50 _= 0.12%. The MIC_50 _of geraniol for *C. tropicalis *was 0.03% and for *C. glabrata *and *C. guillermondii *was 0.06%. Eugenol and linalool were also very active against *C. glabrata*, *C. guillermondii *and *C. tropicalis *with MIC_50 _= 0.06%. Furthermore, terpinen-4-ol was also active against *C. tropicalis *with (MIC_50 _= 0.06%), and *C. glabrata *and *C. guillermondii *(MIC_50 _= 0.12% for both species). In addition, carvacrol was also effective against *C. glabrata *and *C. tropicalis *(MIC_50 _= 0.12% for both species) and menthol against *C. glabrata*, *C. guillermondii *and *C. tropicalis *with MIC_50 _= 0.12%. The MIC_50 _of menthone for *C. tropicalis *was 0.06% and for *C. glabrata *was 0.12%. These compounds were active against those species such as *C. glabrata *and *C. krusei *including isolates categorized as susceptible-dose dependent (2 or 5 out of 10 *C. glabrata *at 24 or 48 h, respectively) and resistant (1 *C. krusei*) to fluconazole.

Farnesol and tyrosol were the less potent evaluated compounds with range of MICs at 24 h of 0.12-8% and 0.5-8% respectively being farnesol more active against *C. glabrata *(MIC_50 _= 0.25%) and *C. dubliniensis *(MIC = 0.25%).

MFC_90 _values of the 10 tested compounds and fluconazole are also summarized in Table 2. Those compounds with more fungicidal activity against *C. albicans *were geraniol, carvacrol, eugenol, linalool and terpinen-4-ol whose MFC_90 _ranged between 0.25 to 0.5%.

## Discussion

Essential oils are very complex natural mixtures extracted from several aromatic plants which can contain more than 20-60 components at quite different concentrations. The components are characterized by low molecular weight and include two groups of distinct biosynthetic origin. The main group is composed of terpenes and terpenoids. The second group includes aromatic and aliphatic products [8,9]. Terpenes are made from combinations of several 5-carbon-base units called isoprene. The monoterpenes are formed from the coupling of two isoprene units. They are the most representative molecules constituting 90% of the essential oils and allow a great variety of structures. Among the monoterpenes, geraniol and linalool are acyclic alcohols; menthol, terpinen-4-ol and μ-terpineol monocyclic alcohols; menthones are monocyclic ketones; and carvacrol and thymol are phenols. The sesquiterpenes are formed from the assembly of three isoprene units, being the farnesol an alcohol [8]. The aromatic compounds such the eugenol, derived from phenylpropane, occur less frequently than the terpenes. Tyrosol is an antioxidant derivative of phenethyl alcohol present in a variety of natural sources such as the olive oil.

Many diverse activities of essential oils and their components have been shown such as antibacterial, antifungal, antioxidant, antitumor, analgesic and antioxidant activities [14]. Their antifungal properties are related to terpenes ability to pass through the fungal cell wall and locate between fatty acid chains of lipid bilayers, disrupting lipid packaging and altering the structure of the cell membrane [15,16]. Braga and Del Sasso [15] demonstrated by scanning electron microscopy that thymol affected the envelope of planktonic *C. albicans*. Changes in permeability and in membrane fluidity cause degradation of cell wall, a decrease in adherence to host's surfaces and variable effects such as disruption of cytoplasm membrane, leakage of cell contents, coagulation of cytoplasm and cell lyses [8,9,14,17].

Compounds, such as carvacrol and geraniol, containing oxygen are frequently considered as those major responsible for this effect [8]. In some compounds, such as carvacrol and thymol, the mechanisms of action seem to be related to the inhibition of ergosterol biosynthesis [18].

When evaluating the antimicrobial properties of oils or their components, the method for determining MIC is important, in order to permit comparison of the data generated by different laboratories. For this reason, CLSI reference method for antifungal susceptibility testing was used in the current study [12], fluconazole was considered as comparator and the potential antifungal activity of almost all the tested components of the essential oils was observed. Eight of ten natural products: carvacrol, eugenol, geraniol, linalool, menthol, menthone, terpinen-4-ol, and α-terpineol showed MICs ranged between 0.03% and 4%. Conversely, farnesol and tyrosol showed lower antifungal activities with MICs ranging between 0.12% and ≥ 8%. These latter compounds have been recognized as quorum-sensing molecules involved in the coordination of activities among groups of many single-celled organisms [19,20]. Farnesol and tyrosol are produced by *C. albicans *which block and accelerate, respectively, the morphological transition from yeasts to hyphae, and are important molecules in different steps of biofilm development and dispersion to other candidal foci during colonization and infection [19,20]. Their weak antifungal activities have been previously described by several authors [11,16]. However, He et al. [21] and Dalleau et al. [11] observed the high efficiency of some terpenes, such as carvacrol, eugenol geraniol and thymol, against *C. albicans *planktonic and sessile (biofilm) cells.

The phenylpropanoid eugenol and the oxygenated monoterpenes geraniol, linalool and terpinen-4-ol were the compounds with the strongest antifungal activity against *C. albicans*. Other authors have observed the potent antifungal or even fungicidal effects of α-terpineol and terpinen-4-ol [17,22,23].

Carvacrol is an important component of several essential oils from plants, such as *Origanum *spp., *Satureja hortensis*, *Thymus *spp. and *Thymbra capitata*. In agreement with our observations, other authors have found that this compound exhibited a potent anti-*Candida *activity [11,24] even against fluconazole-resistant *Candida *isolates. In the current study, carvacrol and the rest of tested compounds, with the exception of farnesol, menthone and tyrosol, showed important in vitro activities at very low concentrations against the fluconazole-resistant *C. krusei *and the susceptible-dose dependent *C. glabrata *isolates. Moreover, carvacrol was able to inhibit in vitro germ tube formation and filamentation of *C. albicans *[8]. Tampieri et al. [16] considered that carvacrol and geraniol were the most active against *C. albicans *as a concentration of 100 ppm of these compounds inhibited the growth of the unique strain tested.

Some studies have shown that primary monoterpenic alcohols (geraniol and citronellol) have more potent antifungal activity than tertiary alcohols (linalool). Linalool activity is controversial as some authors consider this compound inactive while others found a moderate antifungal activity [8,16]. However, in the current study, we have observed a strong fungicidal activity in both geraniol as linalool (MFC_90 _0.25% for geraniol and 0.5% for linalool).

Mondello et al. (2006) reported that terpinen-4-ol (main component of *Melaleuca alternifolia *-tea tree-oil) was fungistatic (MIC_90 _of 0.06%) and fungicidal (MFC_90 _of 0.125%) against fluconazole-susceptible and resistant *C. albicans *isolates. These authors suggested that this compound could be the mediator of the in vivo activity of tea tree oil in a rat model of vulvovaginal candidiasis. Similar antifungal activities of terpinen-4-ol has been observed in the present study, and MIC ranged from 0.06-0.12% for *C. parapsilosis *and *C. tropicalis*, to 0.06-0.5% for *C. albicans*. Finally, the phenylpropanoid eugenol, main component of cinnamon and clove oils, as in our study, is considered to have a potent anti-*Candida *activity [8,25]. We should stress that there are important difficulties to compare the current results with those reported by other authors due to the different in vitro susceptibility tests used and the potential variation of susceptibilities among the isolates and strains studied. Moreover, many studies have tested *C. albicans *or a very limited number of fungal species or even a unique isolate of each species [16,26,27].

On the other hand, the topical use of these naturally occurring plant active principles is relatively safe, and their side effects reported in the literature are minor, self-limiting and occasional [14,24,28,29]. Terpenes have the potential to be toxic if ingested at higher doses and can also cause skin irritation at higher concentrations. However, low doses of carvacrol or terpinen-4-ol did not induced toxicity for the mucosa in rat models of *Candida *vaginitis [24,28]. Moreover, a big advantage of essential oils and their components is the fact that they are usually devoid of long-term genotoxic risks [14,29].

## Conclusions

Some natural compounds from essential oils, such as carvacrol, eugenol, geraniol, linalool and terpinen-4-ol were active in vitro against a collection of oral *Candida *isolates. Their fungistatic and fungicidal activities might convert them into promising alternatives for the topic treatment of oral candidiasis and *Candida*-associated denture stomatitis. However, potential intolerance and/or toxic effects of some of these compounds should be taken into consideration.

## Competing interests

The authors declare that they have no competing interests.

## Authors' contributions

CMA, EE and GQ designed the study. CMA, LM and EE collected the data. CMA and EE performed the techniques employed in the study. CMA, EE and LM made the interpretation of statistical analyses. EE and GQ wrote the paper with input from all the authors who each approved the final version.

## Pre-publication history

The pre-publication history for this paper can be accessed here:

http://www.biomedcentral.com/1472-6882/11/119/prepub
